# Hidalgo Fishes: Dataset on freshwater fishes of Hidalgo state (Mexico) in the MZNA fish collection of the University of Navarra (Spain)

**DOI:** 10.3897/zookeys.403.7149

**Published:** 2014-04-17

**Authors:** David Galicia, Griselda Pulido-Flores, Rafael Miranda, Scott Monks, Ana Amezcua-Martínez, María Imas-Lecumberri, Angel Chaves-Illana, Arturo H. Ariño

**Affiliations:** 1 University of Navarra, Irunlarrea 1, 31008, Pamplona, Spain; 2 University of the Hidalgo State, Centro de Investigaciones Biológicas, Ciudad del Conocimiento, 42184, Pachuca, Mexico

**Keywords:** Occurrence, biometry, freshwater fishes, non-native species, threatened species, conservation, Metztitlán Canyon Biosphere Reserve, Tecocomulco Lake, Mexico

## Abstract

The state of Hidalgo (Mexico) is an important region from the point of view of biodiversity. However, there exists a significant gap in accessible knowledge about species diversity and distribution, especially regarding to freshwater ecosystems. This dataset comprises the sampling records of two projects developed in Hidalgo between 2007 and 2009 about the freshwater fish communities of Tecocomulco lake and rivers belonging to the Metztitlán Canyon Biosphere Reserve. It contains the taxonomic identity (species level) and basic biometric data (total length and weight) as well as date of collection and coordinates of more than 9000 specimens. This dataset is the primary result of the first and unrepeated exhaustive freshwater fish’s survey of Metztitlán Canyon Biosphere Reserve and Tecocomulco lake. It incorporates seven more species to the regional fish fauna, and new exclusive biometric data of ten species. This dataset can be used by studies dealing with, among other interests, North American freshwater fish diversity (species richness, distribution patterns) and biometric analyses, useful for the management and conservation of these areas. The complete dataset is also provided in Darwin Core Archive format.

## Introduction

Fauna and flora of Mexico is significant because of its substantial range of climatic conditions. High diversity of freshwater fish is derived from broad transition between temperate and neotropical biota. Of the 504 species known from the country, ca. 271 are endemic (ca. 48 endemics are from binational basins), 168 are at some level of risk, and 25 are believed to be extinct (Contreras-Balderas et al. 2008; Jelks et al. 2008). The fish fauna of Mexico is highly varied, and its complexity and high rate of endemism are the result of a complex orography, hydrography, and diverse climates (Contreras-Balderas et al. 2008).

Hidalgo is a state in the central area of Mexico and an important region from the point of view of biodiversity of freshwater fishes (Miller et al. 2005). However, there is little information on the continental fishes of this area, and significant increase in support and development of research programs are necessary for the region (Pulido-Flores et al. 2008).

This dataset collection contains the sampling records of two projects about the fish communities of Tecocomulco lake and rivers belonging to the Metztitlán Canyon Biosphere Reserve, developed in 2007–2009 in this state.

The Metztitlán Canyon (Barranca de Metztitlán) Biosphere Reserve, in the northern part of this state, has a high level of endemism in plants and animals because of its geomorphologic origin (Monks et al. 2005). This dataset is the primary result of the first and unrepeated exhaustive freshwater fish’s survey of this Biosphere Reserve, adding seven more species to the regional fish fauna, and new exclusive biometric data of nine species (Miranda et al. 2009, 2012). Among these species, there are five exotic species. Future Biosphere Reserve’s management plans should consider the presence of these alien species, with the aim to preserve conveniently the biodiversity (Pino-del-Carpio et al. 2011).

Lake Tecocomulco is the only remaining natural water body in the basin of Gran Cuenca del Valle de México (Caballero et al. 1999). The occurrences of freshwater fishes present in this lake included in this dataset comprise the first and largest registered population of Chapultepec splitfin *Girardinichthys viviparus*, a threatened goodeid catalogued as critically endangered by the IUCN (Contreras-Balderas and Almada-Villela 1996). This species show an extremely reduced range of distribution in the Mexican plateau, only known from a few locations near Distrito Federal, Mexico City (Navarrete-Salgado et al. 2004, Sedeño-Díaz and López-López 2009) until this dataset registration.

Knowledge of species occurrences is the first step to manage and conserve the biodiversity and scarce information related to the distribution, abundance and management actions of threatened species hinder the development of adequate conservation strategies (Pino-del-Carpio et al. 2011). This is particularly relevant to conservation of species with restricted distribution ranges and seriously threatened, as the Chapultepec splitfin. The existence of this population could prove to be determinant for the conservation and survival of this species (Miranda et al. 2008).

## Project details

**Project title:** Freshwater fishes of Hidalgo state (Mexico)

**Personnel:** Rafael Miranda (principal investigator, data collector, collector identifier), David Galicia (researcher, data collector, data manager), Griselda Pulido-Flores (researcher), Scott Monks (researcher), Carmen Escala (researcher), Berenice Alemán-García (data collector), Rafaela Escorcia-Ignacio (data collector), Antonio Vilches (data collector), Christian Elizbeth Bautista-Hernández (data collector), Pedro Manuel Leunda (data collector), Sergio Gaspar (data collector), Andrés López-Morales (field guide, data collector), Ana Amézcua-Martínez (curator), María Imas-Lecumberri (curator), Ángel Chaves-Illana (curator) and Arturo H. Ariño (custodian steward).

**Funding:** Project CGL2006-02844/BOS from the Plan Nacional de I+D+I (2004–2007), Dirección General de Investigación, Ministerio de Ciencia e Innovación, Gobierno de España. Regional Development Fund (ERDF), project FOMIXHGO-2005-CO1-1 from CONACYT-FOMIX, Hidalgo, Mexico. Agencia Española de Cooperación Internacional of the Ministerio de Asuntos Exteriores y Cooperación, Gobierno de España (A/6357/06).

**Study area descriptions/descriptor:** The state of Hidalgo is located in east central Mexico, at the intersection of the Mexican Neovolcanic Belt, the central highland plateau (Mesa Central) and the Sierra Madre Oriental. Rivers of Hidalgo, part of the Pánuco, Tuxpan and Cazones basins, flow into the Gulf of Mexico. Fishes in the region are relatively diverse and contain Neotropical and Nearctic species, suggesting that this is a transition zone between the two ecozones (Soria-Barreto et al. 1996). The region is characterized by extreme variation in local ecological systems and a high diversity of flora and fauna, produced by geographic isolation of local populations (Pulido-Flores et al. 2005).

**Figure 1.**
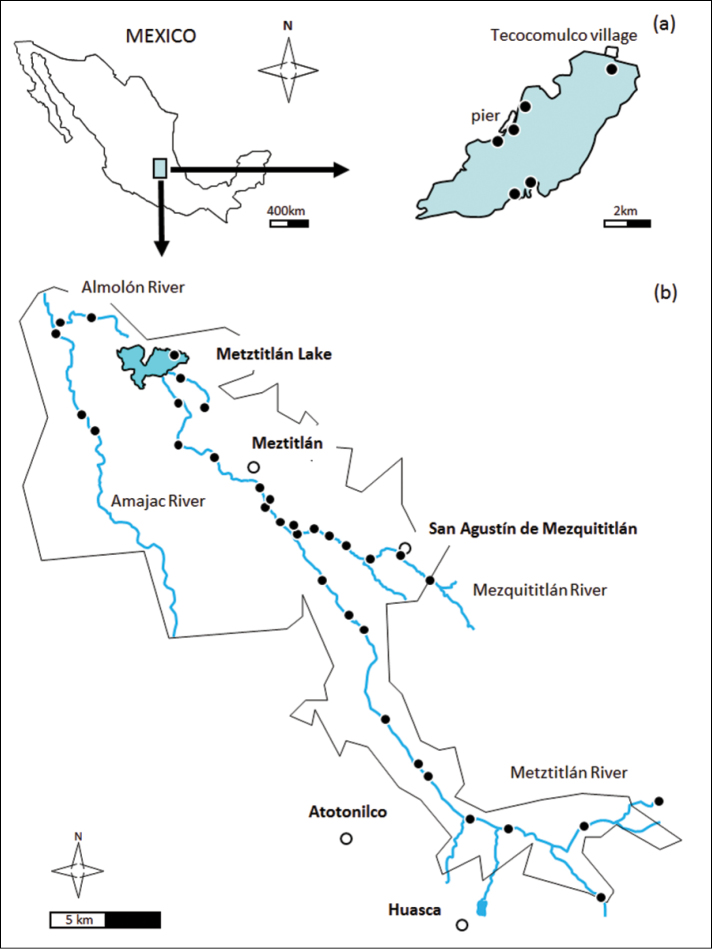
Study area. Locations of sampling points (black dots) in **a** Lake Tecocomulco and **b** Metztitlán Canyon Biosphere Reserve.

The Metztitlán Canyon Biosphere Reserve (Hidalgo, Mexico, Figure 1b) covers an area of approximately 96000 ha and was designated as a biosphere reserve in 2000. The reserve is situated in the rain shadow of the Sierra Madre Oriental, producing an arid climate and receiving just one quarter of the rainfall of nearby areas located at higher elevations within the Sierra Madre Oriental.

Lake Tecocomulco (Figure 1a) is the only remaining natural water body in the basin of Gran Cuenca del Valle de Mexico. Its surface varies from 7 to 15 km^2^, depending on the quantity of seasonal rainfall. The lake has turbid and shallow waters, normally with 15–20 m maximum depth, reaching 3 m during some rainy seasons. Sodium, bicarbonates and sulphates are the dominant ions and smaller proportions of calcium, magnesium and chlorine are present (Caballero et al. 1999). *Scirpus lacustris* L. is the dominant aquatic plant and it extends over much of the central part of the lake. Submerged (*Najas* spp., *Potamogeton* spp.) and free floating (*Lemna* spp.) species are present between the patches of *Scirpus lacustris*.

**Data published through:** GBIF: http://www.gbif.es:8080/ipt/resource.do?r=pemx_mzna

## Taxonomic coverage

**Figure 2.**
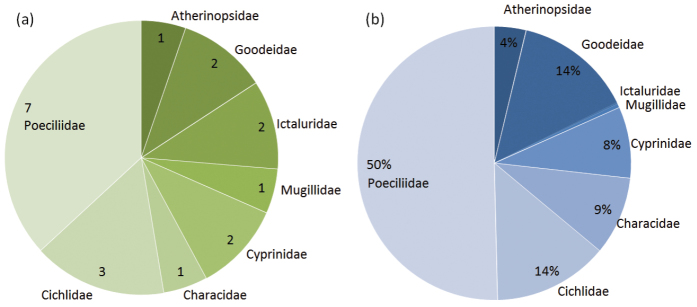
Relative abundance of families. **a** Number of species and **b** percentage of specimens per family recorded in the dataset.

**General taxonomic coverage description:** All specimens are identified to species level with the help of authoritative literature (Hubbs 1924, Hubbs and Turner 1939, Miller 1974, Taylor and Miller 1983, Nelson et al. 2004, Miller et al. 2005). Collection comprises 17 species (and two hybrids) of fishes belonging to eight families of the orders Atheriniformes, Ciprinodontiformes, Ostariophysi and Perciformes. Poeciliidae is the most abundant family, represented by seven species in the HidalgoFFishes dataset, being approximately 50% of the total specimens recorded (Figure 2). Table 1 provides an account of the number of specimens, threatened category according to IUCN red list, ecological affinity and the zoogeographic origin of recorded species. This database includes new records for the State of Hidalgo of the channel catfish *Ictalurus punctatus*, two cichlids (*Herichthys pantostictus* and *Amatitlania nigrofasciata*), two goodeids (*Goodea atripinnis* and *Girardinichthys viviparus*) and three poecilids (*Pseudoxiphophorus jonesii*, *Poeciliopsis gracilis* and *Xiphophorus helleri*). Besides, an undescribed catfish of *Ictalurus* genus has been included on this database (Miller et al. 2005). Among recorded species, there are one species Critically Endangered (*Girardinichthys viviparus*) and other vulnerable (*Herichthys pantostictus*) according to IUCN red list.

**Table 1.** Species and specimens of fish in HidalgoFFishes dataset. Information about threatened category according to IUCN and additional characteristics are provided.

Family	Species	n	Threatened category	Ecological affinity	Zoogeographic origin
Cyprinidae	*Cyprinus carpio*	302			Introduced
	*Tampichthys ipni*	465		Primary	Neartic
Characidae	*Astyanax mexicanus*	843	Least Concern	Primary	Neotropical
Ictaluridae	*Ictalurus punctatus*	1		Primary	Translocated
	*Ictalurus* sp.	18		Primary	Neartic
Cichlidae	*Herichthys pantostictus*	786	Vulnerable A1c, B1+2c	Secondary	Neotropical
	*Amatitlania nigrofasciata*	12		Secondary	Translocated
	*Oreochromis aureus* × *Oreochromis niloticus*	439			Introduced
Atherinopsidae	*Menidia jordani*	342		Peripheral	Translocated
Mugilidae	*Agonostomus monticola*	39	Least Concern	Peripheral	Atlantic
Goodeidae	*Goodea atripinnis*	5	Least Concern	Secondary	Neotropical
	*Girardinichthys viviparus*	1265	Critically Endangered A1ce+2ce, B1+2abc	Secondary	Neotropical
Poeciliidae	*Pseudoxiphophorus jonesii*	390		Secondary	Neotropical
	*Poecilia mexicana*	71		Secondary	Neotropical
	*Poeciliopsis gracilis*	3230		Secondary	Translocated
	*Xiphophorus helleri*	891		Secondary	Translocated
	*Xiphophorus birchmanni*	1		Secondary	Neotropical
	*Xiphophorus birchmanni* × *Xiphophorus malinche*	3		Secondary	Neotropical
	*Xiphophorus malinche*	2		Secondary	Neotropical

## Taxonomic ranks

**Kingdom:** Animalia

**Phylum:** Chordata

**Class:** Actinopterygii

**Order:** Atheriniformes, Ciprinodontiformes, Ostariophysi, Perciformes

**Family:** Mugilidae, Cichlidae, Characidae, Cyprinidae, Goodeidae, Ictaluridae, Atherinopsidae, Poeciliidae

**Species:** *Agonostomus monticola*, *Amatitlania nigrofasciata*, *Astyanax mexicanus*, *Cyprinus carpio*, *Girardinichthys viviparus*, *Goodea atripinnis*, *Herichthys pantostictus*, *Oreochromis aureus* × *Oreochromis niloticus*, *Ictalurus punctatus*, *Ictalurus* sp., *Menidia jordani*, *Poecilia mexicana*, *Poeciliopsis gracilis*, *Pseudoxiphophorus jonesii*, *Tampichthys ipni*, *Xiphophorus birchmanni*, *Xiphophorus birchmanni* × *Xiphophorus malinche*, *Xiphophorus helleri*, *Xiphophorus malinche*

**Common names:** Mountain mullet, Convict cichlid, Mexican tetra, Common carp, Chapultepec splitfin, Blackfin goodea, Chairel cichlid, Channel catfish, NA, Mesa silverside, Shortfin molly, Porthole livebearer, Barred killifish, Lantern minnow, Sheepshead swordtail, Green swordtail, Highland swordtail, Tilapia.

## Spatial coverage

**General spatial coverage:** Hidalgo State, East-Central Mexico. Barranca de Metztitlán Biosphere Reserve (20.23–20.75N; 98.95–98.38W) and Lake Tecocomulco (19.83–19.90N; 98.44–98.35W)

**Coordinates:** 19°49'48"N and 20°45'0"N Latitude; 98°57'0"W and 98°20'60"W Longitude.

### Temporal coverage

The first sample was on February 6, 2007 and the last on November 21, 2008. All the specimens were collected during four two-weeks campaigns that took placed at the beginning and ending of the dry season.

## Natural collections description

Zoological Museum of the University of Navarra (MZNA, Pamplona, Spain) was established in the 1980 to curate the scientific research materials of the former Zoology and Ecology and now Environmental Biology department. Its climate-controlled storage facilities hold more than two million specimens, including several type series.

The Museum is a Data Provider for the Global Biodiversity Information Facility (GBIF) and is an Affiliate to the International Commission of Zoological Nomenclature (ICZN). The Museum is also in charge of the curation and management of the Natural History Collections of the School of Science of the University of Navarra (Spain).

**Parent collection identifier:** 850b564a-f762-11e1-a439-00145eb45e9a

**Collection name:** Peces de México

**Collection identifier:** 28c1c18b-64d8-4691-acdb-73e5653292f8

**Specimen preservation method:** Alcohol

**Curatorial unit:** 6453 with an uncertainty of 0 (observation)

**Curatorial unit:** 950 with an uncertainty of 0 (jar)

## Methods

**Figure 3.**
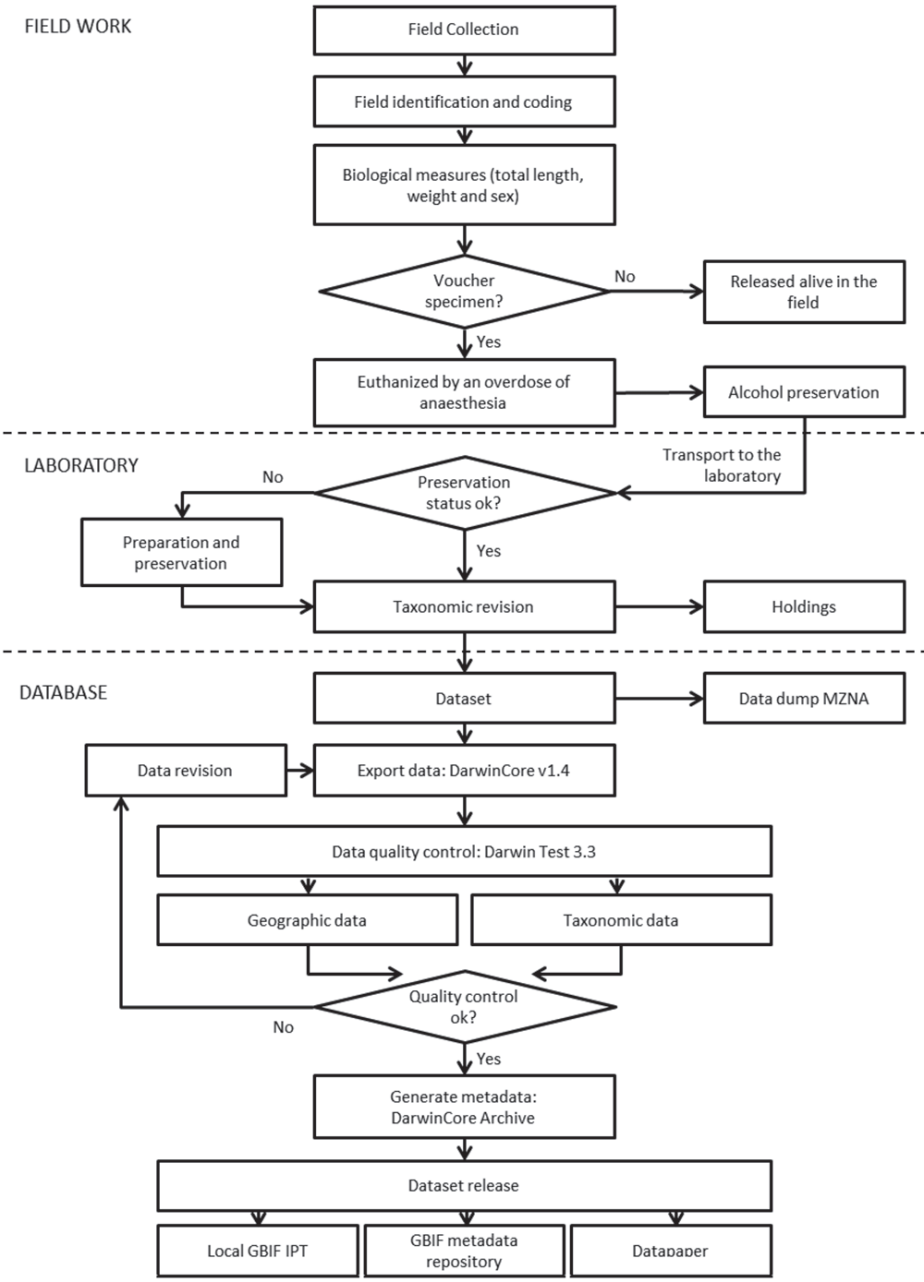
Hidalgo freshwater fishes collection flowchart. The diagram shows all the steps followed from the field sampling to the publishing of the data.

**Method step description:** The processing diagram is shown in Figure 3. Specimens were sampled and processed in the field following the procedure described in the Sampling description section. All the captured specimens where measured, weighted and identified (sex and species) before being released. Some individuals were selected for a deeper study in laboratory and euthanized by an overdose of anaesthesia. Preservation was made directly in the field in 70% ethyl alcohol. Once in the laboratory, all the material was subject of an exhaustive taxonomic revision and field data were corrected accordingly. Project dataset was then incorporated to MZNA database (Zootron v4.5, Ariño 1991), the specimens were then placed in their final containers, consisting on glass jars with 70% ethyl alcohol, labelled properly and deposited in the MZNA museum holdings (except for a subset of individuals that were vouchered elsewhere, see Quality control description section). Dataset was exported to DarwinCore v1.4 format, revised for data inconsistences with DarwinCore standards and corrected if necessary. Once dataset quality was assured, metadata information was added and the derived Darwin Core Archive was incorporated to the Spanish GBIF IPT (http://www.gbif.es:8080/ipt).

**Study extent description:** The state of Hidalgo is located in east central Mexico, at the intersection of the Mexican Neovolcanic Belt, the central highland plateau (Mesa Central) and the Sierra Madre Oriental (Figure 1). Rivers of Hidalgo, part of the Pánuco, Tuxpan and Cazones basins, flow into the Gulf of Mexico. The region is characterized by extreme variation in local ecological systems and a high diversity of flora and fauna, produced by geographic isolation of local populations. The study region is part of the Priorities Hydrologic Region of Mexico because the area is impacted by activities of humans and exhibits high levels of biodiversity (Arriaga et al. 2002). Although this area is important, its ichthyofauna is largely unknown. Some of freshwater fishes included in this dataset are endemics (like the catfish *Ictalurus* sp. or the lantern minnow *Tampichthys ipni*) and threatened (like the Chairel cichlid *Herichthys pantostictus* or the Chapultepec splitfin *Girardinichthys viviparus*). The greatest threats to the long-term existence of these species emerge from agricultural and tourist activities and their effect on the habitat such as exploitation and irrigation for surrounding fields (Miranda et al. 2008).

**Sampling description:** Fourty three localities along the Amajac and Metztitlán rivers and Tecocomulco lake were sampled (Collecting permit SGPA/DGVS/060804/06) using a back-pack electrofishing unit (300-600 V, 0.2-2 A) in November 2007 and May 2008. Fish were anaesthetized with tricaine methanesulfonate (MS-222; Sigma Chemical Co., St. Louis, MO) before being sexed, measured (total length, in mm) and weighed (g). Majority of specimens were returned to their habitat. Voucher specimens were euthanized by an overdose of anaesthesia and transported to the laboratory for taxonomic identification and study (Figure 3).

**Quality control description:** Specimens are deposited in the Zoological Museum of the University of Navarra (MZNA, Pamplona, Spain), in the Colección de la Universidad del Estado de Hidalgo (UAEH, Pachuca, Mexico) and in the Texas A&M University, Rosenthal Lab. (A&M, Texas, EEUU).

The taxonomic identity of all the species and hybrids was verified in the laboratory by R. Miranda and D. Galicia using suitable literature (Hubbs 1924, Hubbs and Turner 1939, Miller 1974, Taylor and Miller 1983, Miller et al. 2005). Scientific names were validated according to W. N. Eschmeyer’s Catalog of Fishes (Eschmeyer 2014).

Unique collections’ accession numbers were assigned to each specimen. Other validation procedures, including geographic coordinates format, and congruence between collection and identification dates were checked with DARWIN_TEST (v3.3, Ortega-Maqueda and Pando 2008) software (Figure 3).

## Datasets

**Dataset description:** Dataset comprises 7403 registries with information of 38 elements of the DwC standard: id, modified, institutionCode, collectionCode, ownerInstitutionCode, basisOfRecord, catalogNumber, occurrenceRemarks, recordedBy, individualCount, sex, preparations, disposition, eventDate, verbatimEventDate, continent, country, stateProvince, locality, verbatimElevation, minimumElevationInMeters, maximumElevationInMeters, verbatimCoordinates, decimalLatitude, decimalLongitude, geodeticDatum, coordinateUncertaintyInMeters, pointRadiusSpatialFit, identifiedBy, scientificName, kingdom, phylum, class, order, family, genus, specificEpithet, scientificNameAuthorship. Also, a Measurement or Facts extension is included with additional biometric information of the specimens: length (total and with sword in the case of swordtail fishes) and weight.

**Object name:** Darwin Core Archive Freshwater fishes of Hidalgo State (Mexico) in the MZNA fish collection of the University of Navarra (Spain)

**Character encoding:** UTF-8

**Format name:** Darwin Core Archive format

**Format version:** 1.0

**Distribution:** http://www.gbif.es:8080/ipt/archive.do?r=pemx_mzna

**Publication date of data:** 2014-01-24

**Language:** English

**Licenses of use:** This dataset [Freshwater fishes of Hidalgo State (Mexico) in the MZNA fish collection of the University of Navarra (Spain)] is made available under the Open Data Commons Attribution License: http://www.opendatacommons.org/licenses/by/1.0/.

**Metadata language:** English

**Date of metadata creation:** 2014-03-25

**Hierarchy level:** Dataset
